# Feasibility, validity and reliability of objective smartphone measurements of physical activity and fitness in patients with cancer

**DOI:** 10.1186/s12885-018-4983-4

**Published:** 2018-10-29

**Authors:** Joeri A. J. Douma, Henk M. W. Verheul, Laurien M. Buffart

**Affiliations:** 1 Department of Medical Oncology, Cancer Center Amsterdam, Amsterdam University Medical Centers (Amsterdam UMC), location VU University Medical Center (VUmc), De Boelelaan 1089a, 1081 HV Amsterdam, The Netherlands; 2 Department of Epidemiology & Biostatistics, Amsterdam Public Health research institute, Amsterdam University Medical Centers (Amsterdam UMC), location VU University medical center (VUmc), Amsterdam, The Netherlands

## Abstract

**Background:**

A patient’s physical function plays a leading role in the treatment prescription for patients with cancer. Objective assessments of physical function may be more predictive for treatment tolerability and survival than frequently used subjective measures, such as the Eastern Cooperative Oncology Group/World Health Organization (ECOG/WHO) performance score. The use of smartphones to measure physical activity and fitness may provide an excellent opportunity to objectively estimate a patient’s physical function against low costs and little time. We investigated feasibility, validity and reliability of smartphone measurements of step count and physical fitness in patients with cancer.

**Methods:**

In total, 72 patients participated. They wore a smartphone for 14 days to measure the mean number of steps per day, concomitant with an accelerometer during the first 7 days. Patients performed a six-minute walk test (6MWT) twice outdoors via a smartphone application and once in a test environment in the hospital. Feasibility was evaluated by the proportion of patients who completed the study as well as smartphone assessments of step count and physical fitness. Validity was assessed with the intraclass correlation coefficient (ICC) between the accelerometer and the first week of the smartphone for step count, and between the 6MWT in the hospital and via the application for physical fitness. Test-retest reliability was assessed with the ICC between step count levels of the first and second week of smartphone assessments, and between the first and second six-minute walk test in the home environment.

**Results:**

The completeness of smartphone measurements was approximately 90% for step count and 64% for physical fitness assessments. Validity was excellent for step count (ICC = 0.97, *p* < 0.001) and fair for fitness (ICC = 0.47, *p* < 0.001). We found excellent test-retest reliability for step count (ICC = 0.91, *p* < 0.001) and physical fitness (ICC = 0.88, *p* < 0.001).

**Conclusions:**

This study showed that objective smartphone measurements of step count in clinical practice are feasible, valid and reliable. These findings indicate that the use of smartphones to objectively assess physical activity in clinical cancer practice is promising and may be used to select patients for treatment and study participation, to monitor patients during treatment and to guide treatment decisions.

## Background

A patient’s physical function plays a leading role in the prescription of treatment for patients with cancer [1, 2]. The physical function of a patient relates to the levels of physical activity and fitness and is in current clinical practice often estimated with the subjective Eastern Cooperative Oncology Group/World Health Organization (ECOG/WHO) performance score [3]. Physical activity is defined as any bodily movement caused by contraction of skeletal muscles resulting in expenditure of energy [4] and physical fitness is defined as a set of health and skill-related attributes that people have, of which cardiorespiratory fitness is one of the main components [4]. Step count, defined as the number of steps per day, is a measure of physical activity, which can easily and objectively be assessed and implemented on a large scale, with low-cost devices (e.g. pedometers) [5]. Maximum oxygen uptake assessed during a maximal exercise test is the gold standard for assessing cardiorespiratory fitness [6]. Unfortunately, the required specialized equipment, well-trained personnel and high costs hamper the implementation on a large scale in clinical practice. A 6 min walk-test (6MWT), which assesses the distance that a person can walk in 6 min, is considered a valid and reliable measurement of cardiorespiratory fitness [7]*.* However, the conduct of a 6MWT requires qualified personnel and an appropriate location inside the hospital, which are both too time consuming and costly in clinical practice [6].

Currently, many people own smartphones which are equipped with advanced technologies (e.g. gyroscope) [8], providing the opportunity for patients to objectively assess their levels of physical activity (step counts) and fitness in their home environment with little time investment and at low costs [9, 10]. In a previous systematic review, it was reported that smartphones have average-to-excellent measurement accuracy in healthy volunteers [11], but were less accurate at slower walking speeds [8]. Objective measurements of physical activity and fitness might improve the estimation of a patient’s physical function [3] and may be useful to optimize treatment selection or to monitor physical activity and fitness levels during and following treatment. Before implementation of objective smartphone measurements in clinical practice, knowledge of feasibility, validity and reliability in patients with cancer, who have lower physical activity levels and reduced physical fitness [6, 12–15], is required.

This study aimed to examine the feasibility, validity and reliability of smartphone measurements of step count and physical fitness in patients with cancer. We hypothesize that the smartphone measurements are feasible, valid and reliable in patients with cancer.

## Methods

### Study design

Patients were recruited from the outpatient Medical Oncology department of Amsterdam University Medical Centers (Amsterdam UMC), location VUmc, and approached consecutively by a researcher (JD). Patients were eligible if they (i) were 18 years or older, (ii) were diagnosed with advanced cancer or received adjuvant (chemo)therapy for localized cancer, and (iii) had a stable performance score. The performance score is a widely used method to assess the functional status of patients with cancer and ranges from 0 (fully active) to 5 (dead), in which 1 reflects a patient who is “restricted in physically strenuous activity but ambulatory and able to carry out work of a light or sedentary nature” and 2 meaning that the patient is “ambulatory and capable of all self-care but unable to carry out any work activities; up and about more than 50% of waking hours” [16]. To exceed the recommended number of 50 patients for validation studies [17], we aimed to include 70 patients. Because earlier studies suggest a low validity of smartphone measurements at slow walking speeds, we aimed to achieve an equal distribution of patients with a different performance score. Consequently, patient enrollment in a specific performance score subgroup stopped after inclusion of 25 patients.

### Measurements

The performance score of the patient had to be scored by the treating medical oncologist before inclusion and needed to be stable, which was defined as the same performance score for two consecutive consultation visits.

Usability and user-friendliness was assessed with the system usability scale (SUS), a 10-item questionnaire designed and validated to assess usability of electronical systems [18]. The questionnaire yields a total score ranging from 0 to 100, for which a ≥ 70 is considered good usability [18].

Physical activity was defined as the mean number of steps per day and was assessed with both an accelerometer (Actigraph wGT3X) and a smartphone (IPhone SE, iOS 10.2). Patients were instructed to wear the smartphone for 14 consecutive days in the hip-waist region, either in a pocket or attached to a belt, during all waking hours, concomitant with a waist-worn accelerometer during the first 7 days.

To calculate the mean daily number of steps per week, at least 4 valid days per week of wearing-time were needed [19]. For the accelerometer, a valid day of wearing-time was defined as 8 h and non-wearing time was defined as 60 min of consecutive zero counts [19]. Raw accelerometer data were processed using ActiLife Software version 6.13.2 (ActiGraph, Pensacola, Florida, USA). Due to the inability to perform a comprehensive analysis of wearing time for the smartphone measurements, we considered every day the smartphone had recorded any steps as a valid wear day. Physical fitness was assessed with the six-minute walk test (6MWT), measuring the maximum distance walked in 6 min [20]. Patients were instructed to perform a 6WMT twice outdoors in their home environment using a smartphone application (Walkmeter), which used the Global Positioning System (GPS)-signal to assess distance. During the same week, a 6MWT was performed under standardized conditions in the hospital [20].

Age, gender, height, weight, zip code, performance score, tumor type, treatment type and treatment intention were retrieved from the medical records. BMI was calculated based on the objective measurements of height and weight (body weight/height^2^, kg/m^2^). Socio-economic status (SES) was determined using zip codes of the patients’ living area [21]. Zip codes were translated to SES according to The Netherlands Institute for Social Research. This system describes the social status of a district compared to other districts in The Netherlands using an algorithm based on mean income, percentage of people with low income, percentage of people with low education, and percentage of people with without a job. Therefore, the mean score of all districts in The Netherlands is zero.

### Statistical analysis

Differences in sociodemographic and clinical characteristics between the participants and non-participants were investigated with univariable logistic regression analyses.

Feasibility was evaluated by the proportion of patients who (i) completed the study, (ii) had ≥4 valid wear days with the smartphone and (iii) completed the 6MWT via the application at least once. Criterion validity of smartphone physical activity assessments was determined by calculating the intraclass correlation coefficient for agreement (ICC) and 95% confidence intervals (CI) between the mean number of steps per day assessed with the smartphone and the accelerometer as reference measure. For physical fitness, we calculated the ICC between the 6MWT performed with the smartphone and the 6MWT performed in the hospital. Bland-Altman plots were used to visualize systematic differences and 95% limits of agreement. Potential proportional bias was quantified with a linear regression analysis between the difference and the mean of both measurements (accelerometer and smartphone). Test-retest reliability of the smartphone’s physical activity and fitness measurements was determined by calculating the ICC, between the mean number of steps during the first and second week and between the first and second 6MWT. An ICC ≥ 0.75 was considered excellent [17, 22]. Standard error of measurement (SEM) and smallest detectable difference at a 95% confidence interval (SDD_95_) were calculated [17]. Sensitivity analyses were conducted to examine validity and reliability separately per performance score.

## Results

**Fig. 1:**
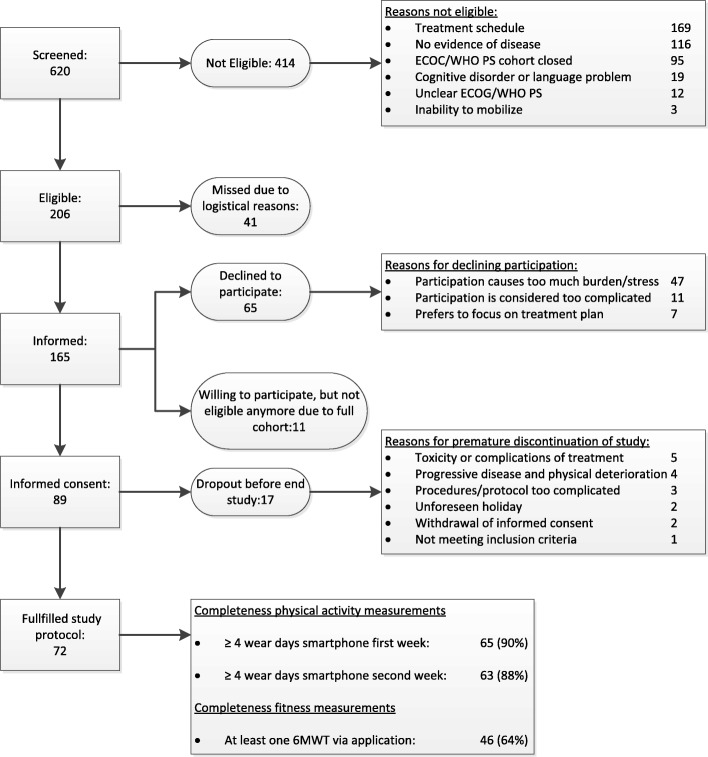
Flowchart of patient inclusion and feasibility of assessments. Legend: ECOG/WHO PS = ECOG/WHO performance score, 6MWT = six-minute walk test

Mainly due to toxicity and/or complications of treatment and progressive disease, 17 of 89 eligible patients (19%) dropped out (Fig. 1). Baseline characteristics of participants and non-participants are presented in Table 1. The proportion of men was 63% amongst the participants and 45% amongst the non-participants (*p* = 0.04). No other statistically significant differences in sociodemographic and clinical factors between the participants and non-participants were found. The proportion of patients with ≥4 valid wear days with the smartphone was 90% in the first and 88% in the second week. At least one 6MWT was performed via the application by 64% of patients. Of the 25 patients who did not successfully complete a 6MWT, 15 did not report a specific reason for the missing data, 4 patients forgot to perform a 6MWT, and 6 patients performed a 6MWT but either encountered a technical problem of the application (*n* = 3), had a poor GPS-signal (*n* = 2), or accidently erased the results (*n* = 1). The mean (SD) score of the SUS was 69 (17).

**Table 1:** Baseline characteristics of participants

	Participants	Non-participants	*P*-value
Gender, No. (%) men	45 (63)	29 (44.6)	0.04
Age, mean (SD, range) years	63 (11.5, 24–86)	65 (11.7, 26–80)	0.29
BMI, mean (SD) kg/m^2^	25.5 (4.5)	26.2 (5.6)	0.43
Socioeconomic status, mean (SD) score	0.44 (1.08)	0.23 (1.00)	0.24
EORTC-QLQ-C30 physical function, mean (SD) score	78 (21.7)		
ECOG/WHO performance score (PS), No. (%)			0.24
0	25 (35)	23 (35)	
1	25 (35)	29 (45)	
2	22 (31)	13 (20)	
Tumor type, No. (%)			0.73
Colorectal	16 (22)	12 (19)	
Melanoma	13 (18)	21 (32)	
Breast cancer	13 (18)	8 (12)	
Prostate cancer	6 (8)	5 (8)	
Gastric cancer	4 (6)	0 (0)	
Pancreatic cancer	4 (6)	0 (0)	
Other	15 (21)	19 (29)	
Current active treatment type, No. (%)^a^			0.17
Chemotherapy	43 (61)	32 (49)	
Targeted therapy	25 (35)	3 (5)	
Immunotherapy	12 (17)	21 (32)	
Hormonal therapy	4 (6)	1 (2)	
No therapy	4 (6)	3 (5)	
Localized cancer treated with curative intention, No. (%)	5 (7)	7 (11)	0.43

*BMI*body mass index, *EORTC-QLQ-C30* European Organisation for Research and Treatment of Cancer-Quality-Quality of Life Questionnaire-30 item module, *ECOG/WHO* Eastern Cooperative Oncology Group/World Health Organization ^a^: Due to combination of therapies, the total percentage exceeds 100%

**Fig. 2:**
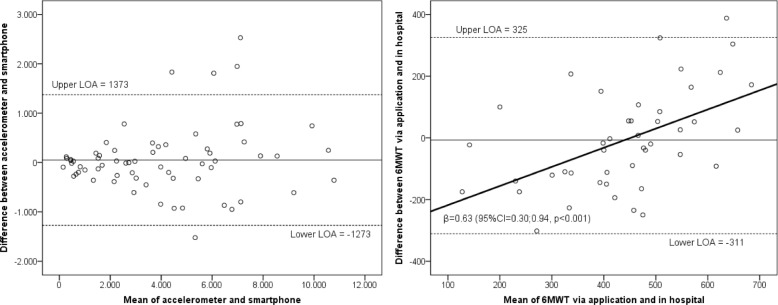
Bland-Altman plots for physical activity and fitness. Legend: β = regression coefficient, LOA = 95% limits of agreement.

The smartphone’s validity was excellent for step count (ICC = 0.97, 95% CI = 0.95–0.98, *p* < 0.001) and fair for fitness (ICC = 0.47, 95% CI = 0.21–0.67, *p* = 0.001) (Table 2). For step count, there were no signs of systematic differences between the measurement with smartphone and accelerometer (Fig. 2). A larger difference between the 6MWT in the hospital and the 6MWT assessed via the application was found in patients with longer walking distances, in favor of the latter (regression coefficient = 0.62, 95% CI = 0.30;0.94, *p* < 0.001) (Fig. 2).

**Table 2:** Construct validity and test-retest reliability for smartphone measurements of physical activity and fitness

Validity physical activity (*n* = 64)	
Accelerometer, mean number of steps/day (SD)	4057 (2883)
Smartphone 1st week, mean number of steps/day (SD)	4033 (2842)
ICC (95% CI, p)	0.97 (0.95–0.98, < 0.001)
Validity physical fitness (*n* = 45)	
6MWT hospital, mean distance walked, in meters (SD)	424 (126)
6MWT via application, mean distance walked in meters (SD)	431 (191)
ICC (95% CI, p)	0.47 (0.21–0.67, 0.001)
Test-retest reliability physical activity (*n* = 61)	
Smartphone 1st week, mean number of steps/day (SD)	4033 (2842)
Smartphone 2nd week, mean number of steps/day (SD)	4414 (2754)
ICC (95%CI, p)	0.91 (0.85–0.94, < 0.001)
SEM	833
SDD_95_	2309
Test-retest reliability physical fitness (*n* = 26)	
1st 6MWT via application, mean distance walked, in meters (SD)	408 (209)
2nd 6MWT via application, mean distance walked, in meters (SD)	478 (182)
ICC (95% CI, p)	0.88 (0.74–0.94, < 0.001)
SEM	70
SDD_95_	193

*SD* standard deviation, *ICC* intraclass correlation coefficient, *CI*confidence interval, p = significance, 6MWT = six-minute walk test, SEM = standard error of measurement, SDD_95_ = smallest detectable difference with 95% confidence

Test-retest reliability was excellent both for step count (ICC = 0.91, 95% CI = 0.85–0.94, *p* < 0.001) and for physical fitness (ICC = 0.88, 95% CI = 0.74–0.94, *p* < 0.001) (Table 2). SEM and SDD_95_ were 833 and 2309 respectively for step count and 70 and 193 for physical fitness (Table 2). Results of the sensitivity analyses across different performance scores were comparable.

## Discussion

The results of this study showed excellent test-retest reliability of smartphone measurements of step count and physical fitness. Feasibility and validity for step count measurements were excellent, but in its present form, they were fair for physical fitness. To the best of our knowledge, this is the first study to investigate feasibility, validity and reliability of smartphone measurements in patients with cancer.

The high validity of smartphone measurements of step count is in line with studies that investigated activity trackers [11, 23] and smartphones [8] in healthy populations. In contrast to results for activity trackers that showed strong dependency on walking speed [23], our sensitivity analyses indicated that results were consistent across different performance scores. The excellent test-retest reliability of smartphone assessments of step count and physical fitness has not been described previously. However, our results indicated that a minimal difference of 2300 steps for step count and 190 m for physical fitness between two measurements is required to detect a real change over time with 95% certainty. It is yet unclear whether this yields sufficient responsiveness to change in clinical settings.

For physical fitness, fair validity and broad limits of agreement indicate that implementation of the 6MWT smartphone application in clinical practice in its present form may be limited. Possible reasons are discouraging user-friendliness of the application and problems with the GPS-signal. Despite relatively low completeness of measurements of physical fitness with the current application and the fair usability of the application, we believe that physical fitness assessments with a customized application might be promising for use in clinical practice.

Strengths of this study are the sufficient sample size and the inclusion of patients with various performance scores, which allows us to draw conclusions about feasibility, validity and reliability of smartphone measurements in patients with cancer with both good and poor physical function. However, men were more likely to participate in the study than women, which may be related to a higher interest in technical gadgets [24]. A limitation of this study is that step count was used as a measure of physical activity, which could lead to an underestimation of total physical activities as it is unable to measure activities such as cycling and swimming [25]. However, both the accelerometer and smartphone assessments of step counts are prone to this underestimation and it is therefore unlikely that it has affected the validity and reliability estimates of step counts. Another limitation is the relatively high number of patients who did not complete a 6MWT via the application. This may limit the implementation of smartphone measurements in clinical practice, but is less likely to affect the results on validity and reliability of smartphone measurements. Our results indicate that technical improvements of the application (e.g. reminders, GPS-processing) could further improve data collection.

Excellent validity and reliability of smartphone measurements of step count support implementation in clinical practice. Furthermore, the results of this study provide evidence that smartphone measurements are feasible and well tolerated in patients with cancer in clinical practice. However, it remains unclear whether objective assessments of physical activity and fitness with smartphones has added value in clinical practice. Therefore, we have initiated a prospective study, aiming to investigate whether smartphone measurements of step count and physical fitness are predictive for trial feasibility in patients with cancer participating in phase I-II clinical trials (NCT03493672). Additionally, it must be verified in future studies whether objective assessment of physical activity and fitness in clinical practice may support timely referral to exercise or rehabilitation interventions.

## Conclusion

In conclusion, the results of this study show that smartphones provide feasible, valid and reliable objective assessments of step count in patients with cancer. For physical fitness, reliability of smartphone measurements was excellent, but in its present form its feasibility and validity was fair. The use of smartphones to objectively assess physical activity and fitness in clinical (cancer) practice is promising.
